# Systemic inflammation and endothelial dysfunction influence the risk and severity of hemorrhagic transformation after endovascular treatment for acute ischemic stroke

**DOI:** 10.3389/fneur.2026.1913218

**Published:** 2026-08-03

**Authors:** Kattalin Álvarez-Agoues, Patricia de la Riva, Jon Rodríguez-Antigüedad, Virginia Gomez, Gorka Arenaza-Choperena, Ana Gorostidi, Noemí Díez, Ana de Arce, Maite Martínez-Zabaleta, Eñaut Garmendia, Alex Lüttich, Maier Anabitarte, Jose Angel Larrea, Alberto Bergareche, Adolfo Lopez de Munain, Juan Marta-Enguita

**Affiliations:** 1 Neurology Department, Donostia University Hospital, San Sebastian, Spain; 2 Ictus, Biogipuzkoa Health Research Institute, San Sebastian, Spain; 3 Faculty of Health Sciences, Deusto University, San Sebastian, Spain; 4 Neurosciences Department, University of the Basque Country, San Sebastian, Spain; 5 RICORS-ictus, National Institute of Health Carlos III, Madrid, Spain; 6 Neurology Department, Hospital de la Santa Creu i Sant Pau, Barcelona, Spain; 7 Radiology Department, Donostia University Hospital, San Sebastian, Spain; 8 Genomics Platform Biogipuzkoa Research Institute, San Sebastian, Spain; 9 Neurodegenerative Diseases, Biogipuzkoa Research Institute, San Sebastian, Spain; 10 CIBERNED (CIBER, Institute Carlos III), Madrid, Spain; 11 Valdecilla Health Research Institute, IDIVAL, Santander, Spain

**Keywords:** Acute ischemic stroke (AIS), biomarkers, endothelial dysfunction, Endovascular treatment (EVT), Flow-mediated dilation (FMD), Hemorrhagic transformation (HT), homoarginine (Harg), neutrophil-to-lymphocyte ratio (NLR)

## Abstract

**Background:**

Hemorrhagic transformation (HT) is a frequent and serious complication, occurring in up to 40% of cases after endovascular treatment (EVT) for acute ischemic stroke (AIS). Inflammation has been increasingly recognized as a key factor influencing both stroke pathophysiology and post-treatment complications (such as HT) interacting with endothelial dysfunction to exacerbate vascular injury after EVT. The objective of this study is to evaluate whether systemic inflammatory status predicts HT in AIS patients, and its relationship with endothelial biomarkers in the setting of this complication.

**Methods:**

We retrospectively reviewed a prospective cohort of 229 AIS patients treated with EVT. Demographic, clinical, imaging, and laboratory data were collected. Inflammatory markers included white blood cell subsets and indices such as neutrophil-to-lymphocyte ratio (NLR), neutrophil-to-platelet ratio (NPR), systemic immune-inflammation index (SII), and systemic inflammation response index (SIRI). Endothelial function was assessed by flow-mediated dilation (FMD) and circulating homoarginine (HArg), asymmetric dimethylarginine (ADMA), and symmetric dimethylarginine (SDMA). The main outcome was radiological or symptomatic HT, classified according to ECASS criteria.

**Results:**

HT was observed in 92 patients (40.2%), of whom 35 (36.1% of HT and 15.3% of the total) were symptomatic. In multivariate analysis, independent predictors of HT included higher NIHSS at admission, higher plasma glucose at admission, the use of non-aspiration devices, lower pre-recanalization lymphocyte count, higher pre-recanalization SII and higher NLR levels. Among endothelial function markers, HArg correlated with inflammatory markers, ANC (*r* = −0.2) and WBC (*r* = −0.19), and was associated to PH and symptomatic HT, but not with any radiologic HT after AIS.

**Conclusion:**

An altered inflammatory status prior to EVT in AIS patients is associated with an increased risk of developing HT after EVT. Additionally, endothelial dysfunction could participate in the more aggressive forms of this complication.

## Introduction

Acute ischemic stroke (AIS) remains one of the most significant non-communicable diseases worldwide, standing as the second leading cause of mortality (approximately 7 million deaths) according to the Global Burden of Disease (GBD) 2021 report (1).

Currently, reperfusion therapy is the most effective treatment for AIS, which consists of intravenous thrombolysis (IVT) with tissue plasminogen activator (such as alteplase or tenecteplase) and endovascular therapy (EVT) in eligible patients (2).

Hemorrhagic transformation (HT) is a major complication in the progression of AIS and a frequent adverse event following reperfusion therapies. Its incidence in follow-up neuroimaging is as high as 40% (3). When HT leads to neurological deterioration, it is referred to as symptomatic intracranial hemorrhage (sICH), which is associated with high mortality and worse outcomes (4).

Multiple factors have been associated with risk of HT following EVT, including, *inter alia*: epidemiological factors (age, pre-stroke treatment, previous diseases, etc.); characteristics of the infarct (size of ischemic core, severity, etc.); EVT treatment times; and poor collateral status (5). However, the precise mechanisms underlying cerebral bleeding in individual patients are still poorly understood.

Identifying risk factors for HT is crucial to optimizing treatment strategies and minimizing adverse events. Among these potential determinants, inflammation has gained attention as a potential contributor to both ischemic injury and post-stroke complications (6, 7). Inflammatory markers (such as C-reactive protein, calprotectin and interleukin-6) in peripheral blood have been investigated as potential indicators of stroke severity and prognosis (8, 9). Recent studies have also explored the prognostic relevance of inflammatory indices, such as the neutrophil-to-lymphocyte ratio (NLR), the Systemic Inflammatory Index (SII), and the Systemic Inflammatory Response Index (SIRI) (6).

HT has also been linked to reperfusion injury in the setting of blood–brain barrier (BBB) disruption and impaired cerebral blood flow autoregulation, processes in which the cerebrovascular endothelium plays a critical role through the synthesis of nitric oxide (NO), a powerful vasodilator. Genetic variations affecting the NO pathway has been associated with interindividual susceptibility to HT (10). This endothelium-NO pathway is also known to interplay with inflammatory mediators from the earliest stages of the ischemic cascade in AIS and the roles of both pathophysiological pathways could be mutually enhanced to facilitate ischemia–reperfusion injury (10, 11).

Endothelial function can be evaluated through multiple approaches. One common approach is the measurement of flow-mediated dilation (FMD) via brachial ultrasound, which provides an assessment of systemic endothelial function (12). Additionally, several NO-related metabolites, including arginine derivatives such as homoarginine (HArg), symmetric dimethylarginine (SDMA), and asymmetric dimethylarginine (ADMA), have been identified as meaningful biomarkers (13).

Although part of the endothelial function dataset (FMD and endothelial biomarkers) was previously reported in a cohort of 150 EVT-treated AIS patients in the study by de la Riva et al. (12), the present analysis expands on that work and focuses on HT as outcome variable. Specifically, this study includes a larger cohort of 229 patients, and adds a comprehensive panel of inflammatory indices (neutrophil-to-lymphocyte ratio, systemic immune-inflammation index, systemic inflammation response index, platelet-to-neutrophil ratio) that were not evaluated in the prior publication.

The aim of this study is to analyze whether patient’s inflammatory status before and after EVT is associated with the development of HT in AIS patients and to explore the interplay between inflammatory and endothelium biomarkers in this context.

## Materials and methods

The data supporting the study’s findings are available from the corresponding author upon request.

### Study design

This is a retrospective review of a prospective cohort of patients treated with EVT and admitted to the Stroke Unit at Donostia University Hospital (DUH) between March 2018 and August 2020.

### Study population

The study included patients diagnosed with AIS due to large vessel occlusion, as confirmed by emergency CT angiography (CTA), who underwent EVT at DUH during the period from March 2018 to August 2020.

Part of this cohort has been previously described in de la Riva et al., J Stroke Cerebrovasc Dis 2025 (12), although the present study includes additional variables and analyses not reported previously.

The inclusion criteria for this study were as follows:

1. Patients presenting with a disabling focal neurological deficit and AIS with confirmed acute vessel occlusion on CTA, involving the proximal middle cerebral artery (MCA), the terminal internal carotid artery (TICA), or other large vessels including the anterior cerebral artery (ACA), posterior cerebral artery (PCA), basilar artery, vertebral artery, or extracranial carotid artery.
2. Availability of basal neuroimaging (CT and CTA) and a 24-h follow-up CT scan to assess for HT after EVT. Follow-up imaging was performed directly with brain magnetic resonance imaging (MRI). As a result, differentiation between contrast staining and true hemorrhage was based on MRI sequences.
3. Treatment with EVT, with or without prior intravenous thrombolysis (IVT), based on standard clinical criteria.

Between March 2018 and March 2020, patients were included consecutively. During the COVID-19 outbreak (March to June 2020), recruitment continued, but not all eligible patients could be included due to the exceptional clinical circumstances. The exact number of non-included eligible patients during this period was not systematically recorded.

### Descriptive variables

The patients’ data were collected through medical record review and anonymized in a dedicated database. The study included the following demographic variables: age, sex, and cardiovascular risk factors (previously known or diagnosed during admission), such as hypertension, diabetes mellitus, dyslipidemia, atrial fibrillation, prior stroke or transient ischemic attack, pre-stroke modified Rankin Scale (mRS), and prior use of antiplatelet and/or anticoagulant drugs.

Clinical variables included stroke severity by NIHSS (National Institutes of Health Stroke Scale) at admission, site of occlusion (e.g., internal carotid artery, segments of the MCA, ACA, PCA, basilar artery, vertebral artery, and extracranial carotid artery), and stroke etiology classified as atherothrombotic, cardioembolic, lacunar, or undetermined.

Radiological stroke severity previous to recanalization was assessed using the ASPECTS (Alberta Stroke Program Early CT Score) scale.

Variables related to reperfusion treatment included IVT, EVT device (categorized as aspiration, stent-retriever, or a combined technique, according to operator preference and anatomical considerations) and time intervals such as onset-to-groin puncture, door-to groin, door-to-reperfusion, groin to reperfusion and onset to reperfusion times. Recanalization success was assessed using the TICI (Thrombolysis in Cerebral Infarction) scale with grades ranging from 0 to 3 (14). Successful recanalization was defined as a TICI score of 2b, 2c, or 3, indicating reperfusion of more than 50% of the affected vascular territory (15).

### Inflammatory variables

Inflammatory status assessment included pre- and post-recanalization measures of white blood cell count (WBC), absolute lymphocyte count (ALC), absolute neutrophil count (ANC), absolute monocyte count (AMC), and absolute platelet count (APC). Additionally, systemic inflammation markers such as C-reactive protein (CRP), the Systemic Immune-Inflammation Index (SII) and the Systemic Inflammation Response Index (SIRI) were defined as follows: SIRI = neutrophil count x monocyte count/ lymphocyte count, SII = platelet count x neutrophil count/lymphocyte count (6, 16). Other indices, including the neutrophil-to-lymphocyte ratio (NLR) and neutrophil-to-platelet ratio (NPR), were also analyzed. NLR was calculated by dividing the neutrophil count by the lymphocyte count. NPR was calculated by dividing the neutrophil count by the platelet count (17, 18).

For pre-EVT measures, venous blood samples were drawn from all patients in the emergency department before the initiation of any treatment as standard-of-care.

Biochemical parameters, including glucose levels, were analyzed. In addition, a complete blood count was performed to determine neutrophil, lymphocyte, monocyte, and platelet counts using standard laboratory techniques. Blood samples were obtained according to routine clinical practice.

After receiving treatment, new blood samples were taken within the following 24 h. Among other parameters, the following were measured: glucose, total cholesterol, low-density lipoprotein (LDL), high-density lipoprotein (HDL), triglycerides, and CRP. CRP was quantified using a particle-enhanced immunoturbidimetric assay with TRIS buffer and bovine serum albumin as reagent, performed on a Cobas c 701/702 analyzer (Roche Diagnostics, Germany). The remaining biochemical parameters were determined with specific enzymatic/colorimetric kits on the same analyzer. A repeat complete blood count was performed to determine neutrophil, lymphocyte, monocyte, and platelet counts, and coagulation parameters were also reassessed.

In addition, serum samples were obtained from arterial blood extracted immediately following femoral artery puncture during EVT. ADMA, SDMA and HArg, were quantified in these samples and in conditioned cell culture media using an ultra-performance liquid chromatography–tandem mass spectrometry method as described in detail in Supplementary methods. The laboratory performing and analyzing the mass spectrometric measurements was blinded to the clinical status of the patients.

Flow-mediated dilation (FMD) was assessed during follow-up, within the first 72 h after stroke onset, and not at admission.

### Outcome variables

The main outcome was HT, defined according to the European Cooperative Acute Stroke Study (ECASS) radiological classification: hemorrhagic infarction (HI) type 1, isolated petechiae without mass effect; HI type 2, confluent petechiae without mass effect; parenchymal hemorrhage (PH) type 1, homogeneous hyperdensity with minimal mass effect involving <30% of the infarcted area; PH type 2, hyperdensity involving >30% of the infarcted area with definite mass effect, possible intraventricular extension, or hemorrhage outside the infarcted area (4). Secondary outcomes were: Modified Rankin Scale (mRS) at discharge and at 90 days post-stroke, good clinical outcome (GCO; defined as a mRS equal or less than 2) at discharge and at 90 days, futile recanalization [defined as poor 90 days functional status (mRs ≥ 3) despite complete or near-complete recanalization, TICI ≥2b] (6).

Symptomatic hemorrhagic transformation (sHT) was defined as any hemorrhagic transformation associated with neurological deterioration attributable to the hemorrhage, typically reflected by an increase of ≥4 points in the NIHSS or a clear clinical worsening temporally related to the bleeding event. In contrast, asymptomatic HT refers to radiological evidence of hemorrhage without any accompanying neurological worsening (19).

### Statistical analysis

Categorical variables are presented as the number and percentage (%) of cases, while continuous quantitative variables are expressed using their mean and standard deviation (SD) or median and interquartile range (IQR), as appropriate. Normality distribution of the data was assessed using the Kolmogorov–Smirnov test. Variables that did not follow a normal distribution were log-transformed and their distribution was reevaluated before being included in adjusted models. First, the study population was divided into two groups: “HT” (the group with any type of radiological HT) and “no HT.” Within the HT group, a distinction was also made regarding whether the HT was symptomatic or not, and each HT was further classified into ECASS radiological subgroups. Baseline characteristics of the study groups were compared using the Chi-square test for categorical variables, the Student’s T-test for quantitative variables with a normal distribution, and the Mann–Whitney U test for quantitative variables without a normal distribution, ordinal variables or when the assumption of homogeneity of variance between the groups is violated. The relationships between endothelial function and inflammatory markers were assessed using Pearson correlation coefficients.

Independent predictors of HT were identified through binary and multivariate logistic regression analyses, including variables with *p* < 0.05 in the univariate analysis. In a second model, sex and age were forced in addition to significant univariate predictors. To minimize collinearity, one variable was excluded when two were highly correlated. Given the expected intercorrelations among inflammatory and endothelial markers, these variables were entered separately into the multivariate model if appropriate. Arginine is listed as a component of the alteplase commercial product, the compound used for IVT in this study, and thus, patients receiving IVT are expected to have higher levels of HArg. To control for this possible confounding factor, we decided to include IVT in the multivariate regression analysis if HArg was included.

Statistical analysis was conducted using IBM SPSS Statistics, version 26 (Chicago, Illinois, USA). Statistical significance was set at *p* < 0.05.

### Ethical considerations

The study was approved by the Ethics Committee of Gipuzkoa on February 20, 2018 under protocol code PRI-CII-2018-01. Written informed consent was obtained from all patients in the study or their relatives, who gave permission to include their data in the study database in accordance with the Spanish Personal Data Protection law.

## Results

During the study period, 233 patients with ischemic stroke underwent EVT. Two were excluded due to missing laboratory data, one due to abnormal results related to myelodysplastic syndrome, and one due to failure of recanalization treatment caused by vascular access difficulties. Ultimately, 229 patients were included. Baseline characteristics are presented in Table 1.

**Table 1:** Univariate analysis and comparison of demographic, clinical, laboratory, and endovascular treatment variables between groups.

Variable	Total	HT	No-HT	*p*
n (%)	229	92 (40.2)	137 (59.8)	
Demographic and clinical variables				
Age, mean (SD)	75.0 (11.7)	74.6 (11.8)	75.3 (11.7)	0.69
Women, n (%)	113 (49.3)	47 (51.1)	66 (48.2)	0.67
Hypertension, n (%)	138 (60.3)	58 (63)	80 (58.4)	0.48
Diabetes mellitus, n (%)	46 (20.1)	**26 (28.3)**	20 (14.6)	**0.01**
Dyslipidemia, n (%)	92 (40.2)	43 (46.7)	49 (35.8)	0.10
Prior stroke, n (%)	33 (14.4)	14 (15.2)	19 (13.9)	0.69
Known AF, n (%)	88 (38.4)	39 (42.4)	49 (35.8)	0.33
Prior antiplatelet therapy, n (%)	56 (24.5)	**29 (31.5)**	27 (19.7)	**0.04**
Prior anticoagulation, n (%)	47 (20.5)	18 (19.6)	29 (21.2)	0.77
Prior mRankin scale, median (IQR)	1 (0–1)	1 (0–1)	1 (0–1)	0.26
Laboratory data at admission				
Glucose (mg/dL), mean (SD)	133.9 (35.2)	**142.5 (44)**	127.3 (26.3)	**<0.01**
Creatinine (mg/dL), mean	0.99 (0.35)	0.98 (0.37)	1 (0.31)	0.92
Triglycerides (mg/dL), mean (SD)	108.4 (99.3)	103.3 (42.4)	112 (125)	0.54
Total cholesterol (mg/dL), mean (SD)	161 (36.7)	155.6 (34.8)	164.8 (37.7)	0.08
HDL(mg/dL), mean (SD)	48.5 (14.5)	47.1 (16.6)	49.6 (12.7)	0.23
LDL (mg/dL), mean (SD)	95.8 (62.1)	87.9 (30.1)	101.5 (77)	0.12
WBC pre-recanalization (X10^3^/μL),mean (SD)	8.8 (3.7)	8.9 (3.5)	8.7 (3.9)	0.70
neutrophils pre-recanalization (X10^3^/μL), mean (SD)	5.9 (3.6)	6.3 (3.4)	5.6 (3.7)	0.18
lymphocytes pre-recanalization (X10^3^/μL), mean (SD)	2 (1)	1.8 (1)	**2.1 (1.1)**	**0.01**
monocytes pre-recanalization (X10^3^/μL), mean (SD)	0.6 (0.5)	0.6 (0.2)	0.73 (0.6)	0.24
platelets pre-recanalization (X10^3^/μL), mean (SD)	222.2 (74.5)	216.6 (69.3)	225.9 (78.0)	0.36
SIRI pre-recanalization (X10^3^), mean (SD)	2.8 (3.3)	3.3 (4)	2.4 (2.7)	0.06
SII pre-recanalization (X10^6^), mean (SD)	1.0 (1.1)	**1.1 (1.5)**	0.8 (0.8)	**0.01**
NLR pre-recanalization, mean (SD)	4.1 (4.5)	**5.1 (5.5)**	3.5 (3.5)	**0.01**
NPR ratio pre-recanalization, mean (SD)	0.04 (0.02)	0.03 (0.02)	0.03 (0.04)	1.0
Endothelial markers				
HArg (μmol/L), mean (SD)	2.3 (1.2)	2.1 (1.2)	2.4 (1.2)	0.17
SDMA (μmol/L), mean (SD)	0.2 (0.1)	0.2 (0.1)	0.2 (0.1)	0.73
ADMA (μmol/L), mean (SD)	0.5 (0.1)	0.5 (0.1)	0.5 (0.1)	0.39
FMD (%), median (IQR)	3 (1.8–3.6)	3 (1.4–5.2)	3.2 (1–6.7)	0.32
Stroke data and endovascular treatment variables				
NIHSS score, median (IQR)	15 (9–20)	**18 (12–22)**	12 (8–18)	**<0.01**
ASPECTS score, median (IQR)	9 (8–10)	9 (7–10)	9 (8–10)	0.11
Cardioembolic vs. others, n (%)	117 (51.1)	47 (51.1)	70 (51.1)	0.85
Aspiration devices vs. others, n (%)	131 (6)	47 (51.1)	**84 (61.31)**	**<0.05**
Time from onset to groin, mean (SD)	324.7 (271.8)	337.0 (240.2)	316.8 (292.1)	0.62
Door-to-needle time (min), mean (SD)	52.4 (20.9)	51.5 (12.2)	52.9 (24.8)	0.20
Door-to-reperfusion time (min), mean (SD)	177.5 (66.4)	177.2 (53)	177.6 (73.9)	0.97
Groin-to-reperfusion time (min), mean (SD)	52.0 (39.6)	**59.9 (47.1)**	46.9 (33.3)	**0.02**
IVT, n (%)	66 (28.8)	23 (25)	43 (31.4)	0.30
Average hospital stay (days), mean (SD)	10.1 (6.6)	**12.2 (9)**	8.7 (3.6)	**<0.01**
Outcomes				
GCO at discharge, n (%)	76 (33.2)	14 (15.6)	**62 (49.6)**	**<0.01**
GCO at 90 days, n (%)	96 (43.6)	21 (23.6)	**75 (57.3)**	**<0.01**
Futile recanalization, n (%)	111 (48.5)	**62 (67.4)**	49 (35.8)	**<0.01**
Death, n (%)	36 (15.7)	18 (19.6)	18 (13.1)	0.20

HT, Hemorrhagic transformation; No-HT, Non-hemorrhagic transformation; SD, standard deviation; AF, atrial fibrillation; LDL, low-density lipoprotein; HDL, high-density lipoprotein; NIHSS, National Institute of Health Stroke Scale; ASPECTS, Alberta Stroke Program Early CT Score; WBC, white blood cells; NLR, neutrophil-to-lymphocyte ratio; NPR, neutrophil-to-platelet ratio; SIRI, systemic inflammation response index; SII, Systemic Immune-Inflammation Index; HArg, homoarginine; ADMA, asymmetric dimethylarginine; SDMA, symmetric dimethylarginine; FMD, Flow mediated dilation; IVT, intravenous thrombolysis; GCO, good clinical outcome. Bold values indicate statistical significance (*p* value < 0.05).

The mean age of the participants was 75.03 years (SD = 11.7), with 116 male patients (50.7%). Upon admission, the NIHSS median score was 15 (IQR = 11). The occluded artery was distributed as follows: 33 TICA (14.4%), 112 MCA1 (48.9%), 54 MCA2 (23.6%), 5 isolated extracranial carotid artery (2.2%), 2 ACA (0.8%), 3 PCA (1.3%), 13 Basilar artery and (5.7%) and 7 vertebral arteries (3.1%). At discharge, 76 patients (33.2%) were independent in their activities of daily living. This percentage increased to 96 patients (43.6%) at 3 months post-discharge.

For the following analysis, patients were divided into two groups: 92 patients (40.2%) who suffered a radiological HT on follow-up neuroimaging were assigned to the “HT” group, and 137 patients (59.8%) were assigned to the “No HT” group. On the other hand, symptomatic HT was present in 35 patients (38%).

In the overall cohort, the median ASPECTS was 9 (IQR 8–10). When stratified by hemorrhagic transformation, patients with HT had a median ASPECTS of 9 (IQR 7–10), compared with 9 (IQR 8–10) in those without HT, with no statistically significant difference between groups (*p* = 0.11; Table 1). The same analysis was performed to compare ASPECTS between patients with symptomatic HT versus no HT, and no significant differences were observed (*p* = 0.21; Table 2).

**Table 2:** Comparison of demographic, clinical, laboratory, and endovascular treatment variables between symptomatic HT and No-HT patients.

Variable	sHT	No-HT	*p*
n (%)	35 (15.3)	137 (59.8)	
Demographic and clinical variables			
Age, mean (SD)	76.7 (11.1)	75.3 (11.7)	0.6
Women, n (%)	13 (56.5)	66 (48.2)	0.46
Hypertension, n (%)	12 (52.2)	80 (58.4)	0.58
Diabetes mellitus, n (%)	7 (30.4)	20 (14.6)	0.06
Dyslipidemia, n (%)	10 (43.5)	49 (35.8)	0.48
Prior stroke, n (%)	3 (13)	19 (13.9)	0.91
Known AF, n (%)	8 (34.8)	49 (35.8)	0.73
Prior antiplatelet therapy, n (%)	**9 (39.1)**	27 (15.7)	**0.04**
Prior anticoagulation, n (%)	4 (17.4)	29 (21.2)	0.68
Prior Rankin scale, median (IQR)	1 (0.25–1)	1 (0–1)	0.28
Laboratory data at admission			
Glucose (mg/dL), mean (SD)	136 (31.4)	127 (26.3)	0.17
Triglycerides (mg/dL), mean (SD)	94 (45.5)	112 (12.5)	0.51
Total cholesterol (mg/dL), mean (SD)	156.7 (38.9)	164.8 (37.7)	0.36
HDL(mg/dL), mean (SD)	53.3 (23.3)	49.6 (12.7)	0.28
LDL (mg/dL), mean (SD)	84.6 (33.7)	101.5 (77)	0.32
WBC pre-recanalization (X10^3^/μL),mean (SD)	8.8 (3.6)	8.7 (3.9)	0.97
Neutrophils pre-recanalization (X10^3^/μL), mean (SD)	6.2 (3.3)	5.6 (3.7)	0.5
Lymphocytes pre-recanalization (X10^3^/μL), mean (SD)	1.8 (1.1)	2.2 (1.1)	0.18
Monocytes pre-recanalization (X10^3^/μL), mean (SD)	0.56 (0.22)	0.73 (0.7)	0.23
Platelets pre-recanalization (X10^3^/μL), mean (SD)	221.2 (63.1)	226 (77.8)	0.78
SIRI pre-recanalization (X10^3^), mean (SD)	2.6 (3)	2.5 (2.8)	0.79
SII pre-recanalization (X10^6^), mean (SD)	1.1 (1.2)	0.8 (0.8)	0.13
NLR pre-recanalization, mean (SD)	5 (5.6)	3.5 (3.5)	0.08
NPR pre-recanalization, mean (SD)	0.03 (0.02)	0.03 (0.04)	0.86
Endothelial markers			
HArg (μmol/L), mean, (SD)	1.8 (0.8)	**2.4 (1.2)**	**0.04**
SDMA (μmol/L), mean, (SD)	0.2 (0.2)	0.2 (0.1)	0.76
ADMA (μmol/L), mean, (SD)	0.5 (0.1)	0.5 (0.1)	0.45
FMD (%), median(IQR)	2 (1.2–3)	**3.3 (2–6.8)**	**0.05**
Stroke data and endovascular treatment variables			
NIHSS score, median (IQR)	**19 (9.5–22.5)**	12 (8–18)	**<0.03**
ASPECTS score, median (IQR)	8 (9–7)	9 (8–10)	0.21
Cardioembolic vs. others, n (%)	9 (39.1)	70 (51.1)	0.37
Aspiration devices vs. others, n (%)	13 (59.1)	84 (69)	0.37
Time from onset to groin, mean (SD)	417.3 (284.9)	316.8 (292.1)	0.20
Door-to-reperfusion time (min), mean (SD)	194.1 (53.7)	177.6 (73.9)	0.39
Groin-to-reperfusion time (min), mean (SD)	63.9 (50.5)	46.9 (33.3)	0.07
IVT, n (%)	4 (17.4)	43 (31.4)	0.17
Average hospital stay (days), mean (SD)	**11.3 (9.4)**	8.7 (3.6)	**0.02**
Outcomes			
GCO at discharge, n (%)	0 (0)	**62 (49.6)**	**<0.01**
GCO at 90 days, n (%)	1 (4.3)	**75 (57.3)**	**<0.01**
Futile recanalization, n (%)	**19 (82.6)**	49 (35.8)	**<0.01**
Death, n (%)	**12 (52.2)**	18 (13.1)	**<0.01**

sHT, Symptomatic Hemorrhagic transformation group; No-HT, Non-hemorrhagic transformation; SD, standard deviation; AF, atrial fibrillation; LDL, low-density lipoprotein; HDL, high-density lipoprotein; NIHSS, National Institute of Health Stroke Scale; ASPECTS, Alberta Stroke Program Early CT Score; WBC, white blood cells; NLR, neutrophil-to-lymphocyte ratio; NPR, neutrophil-to-platelet ratio; SIRI, systemic inflammation response index; SII, Systemic Immune-Inflammation Index; HArg, homoarginine; ADMA, asymmetric dimethylarginine; SDMA, symmetric dimethylarginine; FMD, Flow mediated dilation; IVT, intravenous thrombolysis; GCO, good clinical outcome. Bold values indicate statistical significance (*p* value < 0.05).

Regarding the specific radiologic HT subtypes across the entire study population, 27 patients (11.8%) presented with HI1, 24 patients (10.5%) with HI2, 16 patients (7.0%) with PH1, and 23 patients (10.0%) with PH2. Therefore, parenchymal hemorrhage was observed in 39 out of the 92 patients with HT (42.4%).

Patients in the HT group were more likely to have been treated with antiplatelet therapy (31.5% vs. 19.7%, *p* = 0.04), and presented with more severe strokes upon admission, with a median NIHSS score 6 points higher (*p* < 0.01). Additionally, patients in the HT group had a higher mean plasma glucose level upon admission (142.5 vs. 127.3, *p* < 0.01), with a greater proportion of diabetic patients (56.5% vs. 43.5%, *p* = 0.01).

Regarding prognostic variables, the average hospital stay was 3,5 days longer in the HT group (12.2 vs. 8.7, *p* < 0.05), and there were significantly fewer patients with GCO at discharge (15.6% vs. 49.6%, *p* < 0.01) and at 90 days post-discharge (23.6% vs. 57.3%, *p* < 0.01) without significant differences in mortality. Furthermore, a higher proportion of patients in the HT group experienced futile recanalization (67.4% vs. 35.8%, *p* < 0.01) compared to the No-HT group.

### Inflammatory markers

Mean time from onset to blood sample extraction in the emergency department (pre-treatment) was 204.5 min (SD = 199.5).

Regarding inflammatory parameters (Table 1), some differences were observed between both groups at admission. Lymphopenia was observed more frequently in patients in the HT group (ALC: 1.8 vs. 2.2; *p* = 0.01). Meanwhile, the SII and the mean NLR were higher in the HT group (SII: 1.2 vs. 0.8; *p* = 0.03; NLR: 5.2 vs. 3.5; *p* = 0.02).

The univariate analysis did not reveal significant differences post-recanalization between the two groups for the analyzed inflammatory markers (Supplementary Table 1).

The multivariate analysis (Table 3) identified several independent predictors of HT. Stroke severity, reflected by a higher NIHSS at admission [OR (95%CI): 1.11 (1.05–1.17); *p* < 0.01], and higher plasma glucose levels at admission [per each 10 mg increase (95%CI): 1.18 (1.05–1.32); *p* < 0.05] showed a strong association with HT. On the other hand, the use of aspiration devices [OR (95%CI): 0.38 (0.18–0.77); *p* < 0.01] was associated with a lower risk of HT. Among inflammatory parameters, lower pre-recanalization ALC [per each 10^3^ increases: OR (95%CI): 0.96 (0.93–0.99); *p* < 0.05], higher pre-recanalization SII [per each 10^6^ increase: 1.35 (1.02–1.80); *p* < 0.05] and higher NLR levels [OR (95%CI): 1.11 (1.02–1.22; *p* < 0.05] were associated to the risk of HT (Table 3).

**Table 3:** Univariate and multivariate logistic regression analysis of factors influencing HT after stroke treated with EVT.

Variable	Univariate OR (95%CI)	*p*	Adjusted OR (95% CI)	*p*	Adjusted OR** (95% CI)	p**
Diabetes mellitus	2.3 (1.19–4.44)	0.01	^	^	^	^
Antiplatelet therapy	1.87 (1.02–3.45)	0.04	1.08 (0.47–2.45)	0.86	1.10 (0.48–2.52)	0.82
Glucose at admission (x each 10 mg/dL)	1.13 (1.05–1.24)	<0.01	1.18 (1.05–1.32)	**<0.01**	1.02 (1.01–1.03)	**<0.01**
NIHSS score (per point)	1.09 (1.04–1.14)	<0.01	1.11 (1.05–1.17)	**<0.01**	1.13 (1.06–1.20)	**<0.01**
Groin to reperfusion time (min)	1.01 (1.00–1.02)	0.03	1.01 (0.99–1.02)	0.05	1.01 (1–1.02)	**0.03**
Aspiration Device	0.51 (0.29–0.89)	0.02	0.38 (0.18–0.77)	**<0.01**	0.32 (0.15–0.68)	**<0.01**
ALC (x each 10^3^/ml)*	0.71 (0.54–0.93)	0.01	0.96 (0.93–0.99)	**0.02**	0.95 (0.91–0.98)	**0.01**
SII (x each 10^6^/ml)*	1.35 (1.04–1.76)	0.02	1.35 (1.02–1.80)	**0.04**	1.36 (1.02–1.82)	**0.03**
Pre-NLR (x each/ml)*	1.08 (1.02–1.16)	0.02	1.11 (1.02–1.21)	**0.02**	1.12 (1.02–1.22)	**0.01**

OR, Odds Ratio; NIHSS, National Institutes of Health Stroke Scale, ALC, absolute lymphocyte count; SII, Systemic Immune-Inflammation Index; NLR, neutrophil-to-lymphocyte ratio. ^: excluded from the model due to collinearity. *Inflammatory markers were entered separately into the multivariate model. **Multivariate model including sex, age and variables with a *p* value <0.05 in the univariate analysis. Bold values indicate statistical significance (*p* value < 0.05).

### Endothelial function and inflammatory markers

FMD was performed in 167 patients (72.9%) and the levels of plasma endothelial markers (HArg, SDMA and ADMA) were measured in 148 (64.6%) of the patients. Losses were due to technical issues and the COVID pandemia period where blood samples could not be processed. The time from stroke onset to arterial blood sample collection was 244.5 min (SD 195).

FMD was successfully measured in 167 patients (72.9%). The mean FMD in the overall cohort was 3% (IQR = 1.8–3.6). When comparing groups in our cohort, patients with hemorrhagic transformation showed a mean FMD of versus those without HT (3% [IQR = 1.4–5.2] vs. 3.2% [IQR = 1–6.7], *p* = 0.32) with no statistically significant differences. However, when specifically comparing patients with sHT to those with no HT, FMD was significantly lower in the symptomatic group (2% [IQR 1.2–3] vs. 3.3% [IQR 2–6.8], *p* = 0.05).

Regarding HT, endothelial markers did not show significant association with radiological HT (Table 1) whereas HArg levels were independently associated with the radiological PH subtype of HT in a univariate analysis (2.43 vs. 1.79, *p* < 0.01) and in a multivariate model including the variables associated with any HT (glucose at admission, NIHSS score, aspiration device) [aOR (95%CI): 0.53 (0.3–0.9), *p* = 0.03]. In this context, when assessing symptomatic HT, we found that patients with sHT exhibited lower pre-recanalization HArg levels [1.8 vs. 2.4, *p* < 0.05] and independently predicted sHT (aOR(95%CI): 0.46 (0.22–0.95), *p* = 0.04) in the multivariate analysis (See main Tables 2, 4).

**Table 4:** Univariate and multivariate binary regression analysis of factors influencing sHT after stroke treated with EVT.

Variable	Univariate OR (95%CI)	*p*	Adjusted OR (95% CI)	*p*	Adjusted OR** (95% CI)	p**
Antiplatelet therapy	2.62 (1.03–6.68)	0.04	3.05 (0.95–9.72)	0.06	3.04 (0.95–9.74)	0.06
NIHSS score (per point)	1.08 (1.01–1.15)	0.04	1.03 (0.95–1.12)	0.41	1.03 (0.95–1.12)	0.42
HArg*	0.08 (0.01–0.93)	0.04	0.03 (0.02–0.72)	**0.03**	0.04 (0.02–0.91)	**0.04**
FMD*	0.31 (0.10–0.98)	<0.05	0.40 (0.11–1.48)	0.17	0.32 (0.08–1.32)	0.12
IVT	0.46 (0.15–1.43)	0.18	0.83 (0.24–2.97)	0.78	0.84 (0.23–3.02)	0.79

OR, Odds Ratio; NIHSS, National Institute of Health Stroke Scale; HArg, homoarginine; FMD, Flow mediated dilation; IVT, intravenous thrombolysis. *log-transformed. **Multivariate model including sex, age and variables with a *p* value < 0.05 in the univariate analysis. Bold values indicate statistical significance (*p* value < 0.05).

We also compared hArg concentrations between patients who received IVT. A total of 66 patients (28.8%) of patients received IVT prior to EVT. Patients treated with IVT [showed higher mean levels of HArg (2.1 (sd = 1.2) vs. 2.4 (sd = 1.2), *p* = 0.17)], however multivariate analysis including IVT did not alter the results of the observed associations.

**Figure 1:**
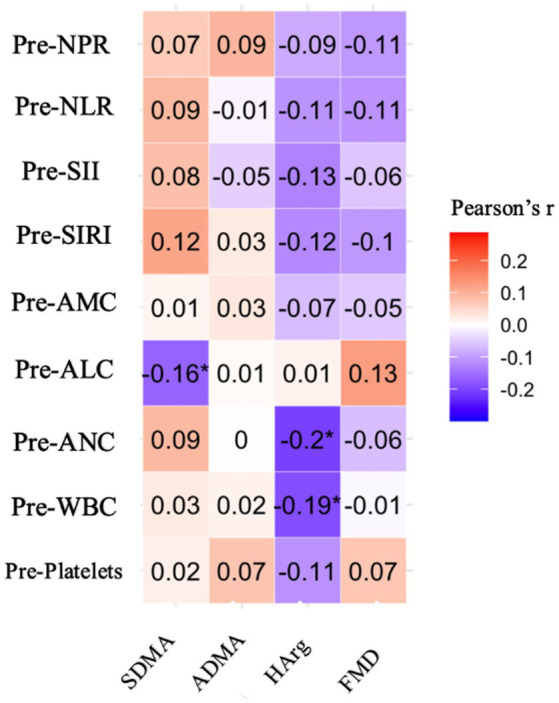
Heatmap of correlations between inflammatory and endothelial function biomarkers. *Indicate *p* < 0.05.

Arginine derivatives showed significant correlations with one another but not with FMD (Figure 1). Correlation analysis between endothelial markers and inflammatory markers revealed pretreatment WBC and ANC correlated with HArg and SDMA correlated with preALC. No correlation was found between systemic endothelial function assessed by FMD and inflammatory markers (Figure 1).

## Discussion

Our findings demonstrate that prerecanalization inflammatory markers, including ALC, NLR and SII, independently predicted radiological HT after EVT indicating that an altered inflammatory status during the acute phase of stroke increases the likelihood of this complication. Moreover, endothelial function markers, particularly HArg, correlated with inflammatory parameters such as ANC and WBC, were associated with PH and sHT suggesting that the endothelium derivatives may contribute to more severe phenotypes of HT.

Inflammation plays a central role in the pathophysiological response to ischemic stroke, exerting both deleterious and reparative effects throughout the disease course. Following cerebral ischemia, innate immune cells (microglia, neutrophils, monocytes) and adaptive immune cells (T cells, B cells, and NK cells) participate in a coordinated response that promotes debris clearance and tissue remodeling. However, these same mediators drive acute peri-ischemic injury through proinflammatory signaling and oxidative stress, ultimately promoting blood–brain barrier (BBB) disruption and HT (20). Neutrophils are a major source of matrix metalloproteinase-9 (MMP-9) in acute ischemic stroke (AIS) and contribute to early BBB damage (21). Conversely, lymphocyte count is a well-recognized marker of physiologic stress; cortisol-driven lymphopenia has been associated with stress-induced immunosuppression and with exacerbation of ischemic damage through cytokine-mediated pathways (22).

The neutrophil-to-lymphocyte ratio (NLR), which integrates both inflammatory activation and stress-related lymphocyte suppression, has been validated as a robust indicator of inflammatory status in AIS, predicting clinical outcomes (6) and the risk of HT (17). Consistent with previous evidence, we observed that higher NLR values (1.11 [1.02–1.22], *p* < 0.05) independently increased the risk of HT.

The systemic immune-inflammation index (SII), which incorporates platelet count and NLR to capture the interaction between thrombosis and inflammation, has similarly been linked to symptomatic HT after EVT (6, 16, 23). This index has also previously been associated with the risk of symptomatic hemorrhagic transformation in stroke patients treated with EVT (6, 16). In this line, our findings support this association, showing that elevated prerecanalization SII values confer a greater risk of HT.

Another pathway involved in peri-ischemic damage and BBB disruption is endothelial dysfunction. Arginine derivatives modulate endothelial homeostasis in cerebrovascular disease: ADMA and SDMA inhibit NO synthase, reducing NO bioavailability and impairing endothelial integrity (13), whereas HArg serves as a substrate for NO production, promoting endothelial health and vascular homeostasis (13). Low HArg levels have been linked to endothelial dysfunction and adverse cardiovascular outcomes, including stroke (13). In the setting of acute ischemic stroke, reduced HArg availability may impair endothelial integrity and regenerative capacity, partly through the diminished function of endothelial progenitor cells (EPC) which play a crucial role in vascular development and reparative responses following vascular injury. Impaired EPC-mediated endothelial recovery exacerbates microvascular instability, facilitating extravasation of blood components into the ischemic parenchyma. Consequently, endothelial dysfunction and insufficient EPC-mediated repair can facilitate hemorrhagic transformation after reperfusion (20).

In our cohort, inflammatory biomarkers showed only modest differences between groups, and their contribution to hemorrhagic transformation appeared limited when compared with other clinical variables such as prior antiplatelet therapy, diabetes, or the type of aspiration device used. Although inflammation demonstrated some significant associations with HT, the overall impact of endothelial dysfunction was small, with homoarginine (hArg) representing the only endothelial biomarker showing significant differences in specific HT subtypes. Therefore, the role of both inflammation and endothelial biomarkers in this context should be interpreted cautiously and considered contributory rather than predominant.

The observation that HArg but not SDMA or ADMA, associated outcomes after stroke may reflect biological differences in their vascular effects, clearance kinetics, and vascular impacts. While ADMA and SDMA act as systemic markers of endothelial dysfunction, HArg may more directly reflect endothelial NO-producing capacity and BBB preservation, explaining its stronger association with HT (13).

The wider IQRs observed in subgroup analyses compared with the overall cohort reflect variability in measurement timing and missing data.

FMD was measured within the first 72 h after stroke onset, a time window in which part of the hemorrhagic transformations may already have occurred. Therefore, FMD should be interpreted as a marker of systemic endothelial status rather than a strict prerecanalization predictor.

Moreover, Harg exhibited significant correlations with inflammatory markers, specifically ANC and WBC, whereas SDMA correlated with preALC. The presence of pro-inflammatory markers in the systemic circulation interferes with vascular function and may exert a deleterious effect on NO bioavailability, representing one of the established links between inflammation and the NO pathway in endothelial cells (11). For instance, elevated CRP levels have been shown to significantly reduce endothelial Nitric Oxide Synthase (eNOS) expression in cultured endothelial cells (22). As reported by Tsikas et al., type 2 diabetes is associated with impaired NO metabolism and altered circulating levels of arginine-derived biomarkers, including ADMA, SDMA and homoarginine (24).

ADMA and SDMA act as mediators of oxidative stress through the inhibition and uncoupling of the NOS enzyme, thereby reducing NO synthesis. On the other hand, inflammation activates arginine methyltransferase proteins (PRMTs) and inhibits DDAHs (dimethylarginine dimethylaminohydrolases), leading to an increase in dimethylarginine, SDMA and ADMA levels (23, 25).

In this context, a correlation between SDMA and inflammatory biomarkers such as IL-6 and monocyte chemoattractant protein-1 (MCP-1) has previously been reported (26). The relationship between HArg and inflammatory biomarkers has not been previously described in the context of stroke, although this molecule may also contribute to oxidative stress.

These findings support the hypothesis that the interaction between inflammation and endothelial integrity not only affects the peripheral vascular bed, but may also exacerbate cerebral ischemic injury, impair collateral perfusion, and limit the efficacy of reperfusion therapies.

Multiple therapeutic approaches under investigation for stroke target inflammatory and NO-endothelium pathways, with varying results. Our study contributes to a more nuanced understanding of the role of the inflammation and NO-endothelium pathways in the context of recanalization therapies and may support the development of better-targeted combination treatment strategies.

This study also expands substantially on our previous work in the same cohort. Compared with the 2025 publication by de la Riva et al. (*n* = 150), which focused on endothelial dysfunction and clinical prognosis, the present analysis includes a larger sample (*n* = 229), incorporates additional inflammatory indices (NLR, SII, SIRI, NPR), and evaluates hemorrhagic transformation as the primary outcome. It also complements the 2022 report on eNOS polymorphisms and HT by integrating endothelial biomarkers (HArg, ADMA, SDMA) with systemic inflammatory status. These additions provide a broader and more integrated view of the inflammation–endothelium axis in EVT-treated stroke and ensure that the present study offers novel and non-overlapping insights (12).

This study has several limitations. First, it was a single-center study with a relatively small sample size, which may restrict generalizability of the findings. Second, although we adjusted for relevant confounders, residual confounding cannot be excluded.

Third, endothelial biomarkers were measured from arterial samples obtained immediately after groin puncture, whereas inflammatory markers were derived from venous blood drawn earlier, with a mean interval of 40 min, which may introduce variability.

These endothelial–inflammatory correlations were weak (r values around −0.2), indicating modest associations rather than strong mechanistic links. In addition, no correction for multiple comparisons was applied, so these exploratory findings should be interpreted with caution.

## Conclusion

Taken together, these data support the hypothesis that the interplay between systemic inflammation and endothelial dysfunction significantly influences the risk and severity of HT following EVT. While radiological HT may occur within a broader inflammatory environment, the development of PH and sHT likely requires a combined burden of inflammatory and endothelial impairment.

## Data Availability

The raw data supporting the conclusions of this article will be made available by the authors, without undue reservation.
